# Bisphenol A Coupled with a High-Fat Diet Promotes Hepatosteatosis through Reactive-Oxygen-Species-Induced CD36 Overexpression

**DOI:** 10.3390/toxics10050208

**Published:** 2022-04-22

**Authors:** Jyun-Lin Lee, Yao-Chien Wang, Yu-An Hsu, Chih-Sheng Chen, Rui-Cian Weng, Yen-Pei Lu, Chun-Yu Chuang, Lei Wan

**Affiliations:** 1 Department of Biomedical Engineering and Environmental Sciences, National Tsing Hua University, Hsinchu 300, Taiwan; jyunlinlee@gapp.nthu.edu.tw; 2 Department of Emergency Medicine, Taichung Tzu Chi Hospital, Taichung 427, Taiwan; j13762135@gmail.com; 3 School of Chinese Medicine, China Medical University, Taichung 404, Taiwan; annhsu007@gmail.com; 4 Division of Chinese Medicine, Asia University Hospital, Taichung 413, Taiwan; g39605020@gm.ym.edu.tw; 5 Department of Food Nutrition and Health Biotechnology, Asia University, Taichung 401, Taiwan; 6 Department of Chinese Medicine, China Medicine University Hospital, Taichung 404, Taiwan; 7 Graduate Institute of Biomedical Electronics and Bioinformatics, National Taiwan University, Taipei 106, Taiwan; cian@itrc.narl.org.tw; 8 National Applied Research Laboratories, Taiwan Instrument Research Institute (TIRI), Hsinchu 300, Taiwan; ypl@tiri.narl.org.tw; 9 Department of Obstetrics and Gynecology, China Medical University Hospital, Taichung 404, Taiwan; 10 Department of Medical Laboratory Science and Biotechnology, Asia University, Taichung 413, Taiwan

**Keywords:** bisphenol A, reactive oxygen species, endocrine-disrupting chemical, CD36, non-alcoholic fatty liver disease, fatty acid uptake

## Abstract

Bisphenol A (BPA) is an endocrine-disrupting chemical that affects lipid metabolism and contributes to non-alcoholic fatty liver disease (NAFLD). The mechanism of BPA exposure in hepatic lipid accumulation and its potential effect on NAFLD remain unclear. This study investigated the effect of BPA-exposure-induced hepatic lipid deposition on the pathology of NAFLD and its underlying mechanism in vitro and in vivo. BPA increased intracellular reactive oxygen species (ROS) levels, and promoted fatty acid uptake through upregulation of a free fatty acid uptake transporter, cluster of differentiation 36 (CD36), in HUH-7 cells. Additionally, C57BL/6 mice administered a high-fat/high-cholesterol/high-cholic acid diet (HFCCD) and BPA (50 mg/kg body weight) for 8 weeks developed a steatohepatitis-like phenotype, characterized by alpha-smooth muscle actin (α-SMA, an indicator of hepatic fibrosis) and cleaved caspase 3 (an indicator of apoptosis) in hepatic tissue; moreover, they had a higher oxidative stress index of 8-hydroxydeoxyguanosine (8-OHdG) in liver tissue compared to the control group. Treatment with ROS scavenger n-acetylcysteine (NAC) ameliorated BPA-mediated HFCCD-induced lipid accumulation and steatohepatitis in the livers of treated mice. Our study indicates that BPA acts synergistically to increase hepatic lipid uptake and promote NAFLD development by stimulating ROS-induced CD36 overexpression.

## 1. Introduction

Non-alcoholic fatty liver disease (NAFLD), the most prevalent chronic liver disease, is characterized by abnormal lipid metabolism [1]. The main causes of NAFLD include poor nutrition and lack of exercise; however, environmental factors may promote obesity and, consequently, the occurrence of NAFLD [2]. Abnormal macrophage infiltration and the associated inflammation can cause fatty liver to develop into irreversible fibrosis, and life-threatening cirrhosis and may lead to hepatocellular carcinoma [3,4]. Homeostasis of hepatic lipids is maintained by hepatocyte uptake and de novo synthesis of free fatty acids (FFAs) [5]. An imbalance between the acquisition and oxidation of FFAs can result in fatty liver or steatosis [6]. Once acquired, FFAs are esterified and stored in hepatocytes as triglycerides (TGs). Although TGs are not intrinsically hepatotoxic, abnormal processing of FFAs by hepatocytes activates resident and infiltrating macrophages through toll-like receptor 4 pathways to initiate a pro-inflammatory cascade and promote the development of NAFLD in mice [7].

Bisphenol A (BPA) is a plasticizer that has long been used in the manufacture of polycarbonate and epoxy resins. BPA has been reported to exhibit estrogenic properties in the reproductive system of female rats [8]. The United States Food and Drug Administration and the European Food Safety Authority have determined that human exposure to BPA should be kept below 50 µg/kg/day. The link between BPA and NAFLD has been reported in several studies [9,10]. For example, BPA can promote lipid accumulation in hepatocytes [11] and increase intracellular TG content in hepatic cells [12]. Chronic exposure to BPA aggravates the development of NAFLD and, in addition to a direct effect of BPA on de novo adipogenesis, the polarization of M1 Kupffer cells is involved in BPA-induced hepatic lipid accumulation [13]. Shimpi et al. found that BPA administration in pregnant CD-1 mice induced Nrf2 expression and recruitment to the sterol regulatory element binding protein 1c (Srebp-1c) promoter, resulting in hepatic lipid deposition [14]. In addition, BPA can act as an E2 (17β-estradiol) mimetic compound by linking to ERα receptors, leading to the increased expression of glucose transporter (GLUT)-4 and glucose uptake [15]. BPA exacerbated hepatic steatosis in OVX mice, which was mediated, in part, by chronic endoplasmic reticulum (ER) stress and the transforming growth factor (TGF)-β1 pathway [16]. The HNF1b/PPARγ pathway is involved in gestational BPA-exposure-induced NAFLD in male offspring mice [17]. However, the pathophysiology of NAFLD remains unclear, and further studies are needed to elucidate the effect of BPA on NAFLD progression.

The abnormal hepatic uptake of lipids under pathological conditions may lead to an excessive accumulation of FFAs and TGs in the liver, causing cytotoxicity and resulting in NAFLD [18]. Endogenous FFAs and nutrients can be internalized by scavenger receptors (SRs). SRs of class A1 (SR-A1) can bind to oxidized low-density lipoproteins (LDLs) and increase their intracellular uptake [19]. SRs of class B1 (SR-B1) are plasma membrane cholesterol sensors with a high affinity for high-density lipoprotein (HDL) cholesterol. Apart from HDL levels, SR-B1 mediates the bidirectional flux of phospholipids and cholesterol between lipoproteins and cell plasma membranes [20]. Cluster of differentiation 36 (CD36) is an SR that facilitates the transport of various lipids, including long-chain fatty acids, phospholipids, and oxidized LDLs [21]. CD36 plays an important role in mediating the production of reactive oxygen species (ROS), regulating the uptake of hepatic fatty acids, and the storage of TGs. The superabundant accumulation of lipids in hepatocytes can cause the oxidative capacity of metabolism to be exceeded, leading to oxidative stress and activation of the TGF-β signaling pathway in liver fibrogenesis; CD36 may participate in this process [22].

In previous reports, the administration of BPA (2.4 µg/kg/day) increased ROS levels in rat livers, decreased antioxidant enzyme activity, and induced chronic inflammation and DNA damage in hepatic tissue [23,24]. In both cell and animal experiments, BPA induced cell toxicity via ROS-initiated ER stress regulated by the eukaryotic initiation factor 2α (eIF2α) and C/EBP-homologous protein (CHOP) pathways [25]. Although ER stress and ROS have been associated with NAFLD, the degree of their contribution to oxidative stress is unclear.

A previous study reported that HepG2 cells and primary hepatocytes treated with the ROS inducer H_2_O_2_ showed higher levels of lipid accumulation compared to untreated cells [26]. However, it is still unclear whether BPA exposure can promote the progression of NAFLD by increasing ROS-induced fatty acid uptake. The present study aimed to investigate whether BPA coupled with a HFCCD promotes the development of fatty liver by enhancing ROS production and lipid accumulation in hepatocytes.

## 2. Materials and Methods

### 2.1. Chemical Reagents and Cell Line

BPA, bovine serum albumin (BSA), sodium oleate (OA), sodium palmitate (PA), n-acetylcysteine (NAC), and phosphate-buffered saline (PBS) were purchased from Sigma-Aldrich (St. Louis, MO, USA). BODIPY FL lactosylceramide (LacCer) complexed to BSA was purchased from Thermo Fisher Scientific (Waltham, MA, USA). The antibodies used included anti-CD36 (GeneTex, Irvine, CA, USA), anti-α-SMA (GeneTex), anti-α-tubulin (GeneTex), anti-collagen I (GeneTex), anti-CHOP (Cell Signaling Technology, Danvers, MA, USA), anti-cleaved caspase-3 (Cell Signaling Technology), anti-SR-A1 (Abcam, Cambridge, UK), and anti-SR-B1 (Novus Biologicals, Littleton, CO, USA).

A human hepatoma HUH-7 cell line was purchased from the Japanese Collection of Research Bioresources Cell Bank (Osaka, Japan). HUH-7 cells were cultured in Dulbecco’s modified Eagle’s medium (DMEM), supplemented with 10% fetal bovine serum (FBS, Sigma-Aldrich) and a 1% mixture of penicillin G, streptomycin, and amphotericin B at 37 °C in a 5% CO_2_ incubator.

### 2.2. Cell Viability Assay

Cell viability was performed using an MTT (3-[4,5-dimethylthiazol-2-yl]-2,5-diphenyltetrazolium bromide, Sigma-Aldrich) assay. Cells (2 × 10^4^) were seeded onto 96-well plates in 0.1 mL growth medium and incubated for 24 h. After exposure to BPA (0, 10, 100, 200, and 400 µM) or BPA plus FFAs (a long-chain fatty acid mixture of OA and palmitate 1:1, 0.5 mM), the cells were treated with MTT; they were incubated at 37 °C for 4 h, the growth medium was removed, and the MTT crystals were dissolved with 1 mL dimethyl sulfoxide (DMSO). Aliquots (100 μL) of the resulting solution were transferred to 96-well plates and optical density was detected at 570 nm within 20 min using a microplate spectrophotometer reader. Cell viability was calculated as a ratio of the optical density for a BPA treatment group divided by that of a vehicle control group.

### 2.3. Intracellular TG Levels and Lipid Staining

We used FFAs at a concentration of 0.5 mM (OA/palmitate, 1:1 equimolar mixture) to induce fat-overloading in cells [27]. After exposure to BPA or BPA plus FFAs for 24 h, cells were washed twice with PBS, fixed with 3.7% formaldehyde in PBS for 30 min, and washed twice with PBS. Intracellular TGs were stained with 0.35% Oil Red O powder (Sigma-Aldrich) in isopropyl alcohol (Sigma-Aldrich) for 30 min. Excess stain was removed by washing with 70% isopropyl alcohol and PBS. The stained lipid droplets were dissolved in isopropyl alcohol containing 4% Nonidet P-40 (Sigma-Aldrich) and quantified at 510 nm on a Synergy HT microplate reader (Biotek, Winooski, VT).

Intracellular lipid content was measured using an AdipoRed assay (Lonza, Allendale, NJ) according to the manufacturer’s instructions. Cells were incubated with 3 mM NAC for 1 h before BPA exposure for 24 h. The stained cells were washed with water, and observed under a fluorescence microscope (Leica DMi8, Leica Biosystems, Wetzlar, Germany). Images were recorded for five different fields of observation and analyzed using ImageJ 1.43u (National Institutes of Health, Bethesda, MD, USA).

### 2.4. Determination of Intracellular Fatty Acid Uptake

To determine fatty acid uptake, we used a fluorescence-labeled long-chain fatty acid probe, BODIPY FL LacCer complexed to BSA (Thermo Fisher Scientific), co-incubated with BPA (0–100 µM) for 6 h. Intracellular uptake of the probe was determined using flow cytometry (BD Biosciences, Franklin Lakes, NJ, USA) and analyzed using FlowJo software (Tree Star, San Carlos, CA, USA).

### 2.5. Measurement of Intracellular ROS

Free radical levels were determined using a DCFDA (2′, 7′-dichlorofluorescin diacetate) assay (Abcam). Cells (2 × 10^4^) were seeded onto a 96-well plate in 0.1 mL of growth medium and incubated for 24 h. Preceding the experiment, the culture medium was removed, and the cells were washed three times with PBS. Cells were incubated for 45 min in Hank’s balanced salt solution (HBSS) containing DCFDA, washed twice, and treated with BPA or BPA plus FFAs for 6 h. Fluorescence was monitored at a 488 nm excitation and a 535 nm emission wavelength using a microplate spectrophotometer reader. Intracellular levels of ROS were determined according to the fluorescence intensity.

### 2.6. Western Blotting and Immunofluorescence

Cells were lysed in lysis buffer (25 mM Tris-HCl (pH 7.6), 150 mM NaCl, 1% NP-40, 1% sodium deoxycholate, 0.1% SDS, and protease and phosphatase inhibitors). The concentration of lysate protein was quantified using a Bio-Rad protein assay (Bio-Rad Laboratories, Hercules, CA, USA) and normalized for loading. Proteins were separated in 8% and 12% SDS-PAGE gels and transferred to a PVDF membrane. After 1 h, the PVDF membrane was blocked with 5% BSA in TBS/TWEEN 20 (TBST) buffer and incubated in 5% BSA/TBST overnight at 4 °C with the primary antibody. The next day, secondary antibody anti-rabbit IgG-HRP (1:5000) or anti-mouse IgG-HRP (1:5000) was used to confirm the primary antibody. The specific proteins were visualized using a chemiluminescence HRP substrate (MilliporeSigma, Bedford, MA, USA) and each band was quantified using a luminescence image analyzer (LAS-4000 mini, Fujifilm Life Sciences, Tokyo, Japan). 

### 2.7. RNA Extraction and Reverse Transcription–Quantitative Polymerase Chain Reaction (RT-qPCR)

Total RNA was extracted using an RNeasy mini Kit (Qiagen, Hilden, Germany) according to the manufacturer’s instructions. cDNA was synthesized using a High-Capacity cDNA Reverse Transcriptase Kit (Applied Biosystems, Foster City, CA, USA). The primer sequences for each gene were as follows: SR-A1 (forward: 5′-TTTGATGCTCGCTCAATGAC-3′; reverse: 5′-TTGAAGGGAAGGGCTGTTTT-3′), SR-B1 (forward: 5′-GGCCTATTCTGAATCCCTGA-3′; reverse: 5′-CTGGCTCACGGTGTCCTC-3′), CD36 (forward: 5′-TCCCAAGCTCAAGTGAATCTC-3′; reverse: 5′-ATGCCAGTTGAATGCCTACC-3′), and GAPDH (forward: 5′-AGCCACATCGCTCAGACAC-3′; reverse: 5′-GCCCAATACGACCAAATCC-3′). The transcript levels of CD36, SR-A1, and SR-B1 were quantified with RT-qPCR using the cDNA as a template in a StepOne Plus system (Applied Biosystems) with universal probes (Roche, Basel, Switzerland). The relative expression of each mRNA was calculated according to 2^−ΔΔCt^ with GAPDH as the internal control.

### 2.8. Animals and Experimental Design

Eight-week-old male C57BL/6 mice (18–20 g) were purchased from the Taiwan National Laboratory Animal Center and National Applied Research Laboratories (NARLabs, Taipei, Taiwan). All surgeries were performed under isoflurane anesthesia. All experimental procedures were approved by the Institutional Animal Care and Use Committee of the China Medical University (CMUIACUC-2018-169-2). After 1 week of acclimation, the initial body weight of each mouse was recorded. The mice were randomly assigned to groups (*n* = 6 per group) according to diet: normal diet (ND, 10% kcal fat), HFCCD (50% kcal fat, 1.25% cholesterol, 0.25% cholic acid) [28], HFCCD supplemented with 50 μg/kg/day BPA (BPA + HFCCD), and BPA + HFCCD + NAC (1 mg/mL NAC dissolved in drinking water). Individual body weight and food intake were measured weekly. No mortalities or side effects were noted during the experimental period. After 8 weeks, mice were euthanized using CO_2_ after fasting for 12 h overnight, and blood and tissue samples were collected. The tissue samples, liver, spleen, and adipose tissue, were weighed on ice immediately after removal and stored at –80 °C. Blood glucose was measured using an Accu-Chek Aviva glucometer (Accu-Chek Advantage, Roche Diagnostic, Mannheim, Germany).

### 2.9. Staining for Fibrosis in Hepatic Tissue

Liver tissues were fixed in 10% neutral-buffered formalin and embedded in paraffin to determine hepatic fibrosis. The tissues were cut into 8-μm sections and placed on slides, followed by staining with hematoxylin and eosin (H&E), Oil Red O, and Masson’s trichrome (Leica Biosystems). For IHC staining, the slides were fixed overnight in 4% paraformaldehyde and PBS and embedded in paraffin. Sections were deparaffinized, immersed in hydrogen peroxide for 30 min, blocked in PBS containing 5% normal goat serum for 1 h at room temperature, incubated overnight at 4 °C with primary antibody anti-collagen I, and incubated with a secondary antibody conjugated to biotin for 1 h at room temperature. The slides used streptavidin-conjugated HRP with diaminobenzidine (DAB; Sigma-Aldrich) as a substrate and were counterstained with hematoxylin. Images were recorded using a fluorescence microscope (Olympus, Tokyo, Japan).

### 2.10. Biochemical Assays

Serum levels of alanine aminotransferase (ALT), aspartate aminotransferase, TG, and total cholesterol were measured using a FUSI DRI-CHEM SLIDE kit with a FUSI DRI-CHEM 4000 analyzer (Fujifilm Life Sciences). Hepatic TG and total cholesterol levels were determined using a Randox kit (Randox laboratories, Kearneysville, WV, USA) according to the manufacturer’s instructions. Plasma insulin levels were determined using a Mercodia kit (Mercodia, Winston-Salem, NC, USA). Hepatic 8-hydroxydeoxyguanosine (8-OHdG) levels were measured using an 8-OHdG ELISA Kit (Wuhan Fine Biotech, Wuhan, China).

### 2.11. Statistical Analysis

Data are presented as the mean ± standard deviation (SD) of at least three independent experiments. Data were analyzed using a Student’s *t*-test or one-way analysis of variance. Statistical significance was set at *p* < 0.05.

## 3. Results

### 3.1. BPA Treatment Induced Cell Death and Increased Intracellular ROS Production

**Figure 1:**
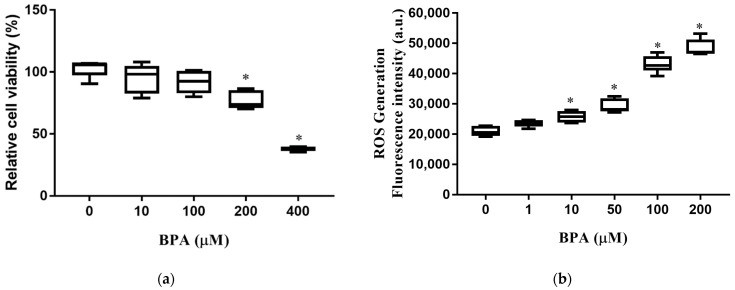
BPA-induced cytotoxicity and ROS production: (**a**) Cells were incubated at different concentrations of BPA for 24 h. Cell viability was measured using an MTT assay; (**b**) cells were pretreated with 2′, 7′-dichlorofluorescin diacetate for 45 min and loaded with BPA for 6 h. Intracellular ROS levels were determined according to fluorescence intensity. The boxplots present the median (central horizontal line), 25th and 75th quartiles (upper and lower limits of the box), and the maximum and minimum range values (error bars). The results are expressed as the mean ± SD of six independent experiments. * *p* < 0.05 vs. control (without BPA) group.

We first determined the effect of BPA exposure on cell viability and intracellular ROS production. The viability of HUH-7 cells decreased significantly with exposure to BPA at concentrations of 200 and 400 µM (Figure 1a). Intracellular ROS production in HUH-7 cells increased in a dose-dependent manner. (Figure 1b). These results indicate that exposure to BPA increases ROS production, leading to cell death.

### 3.2. BPA Treatment Enhanced Accumulation and Uptake of Lipid Droplets

**Figure 2:**
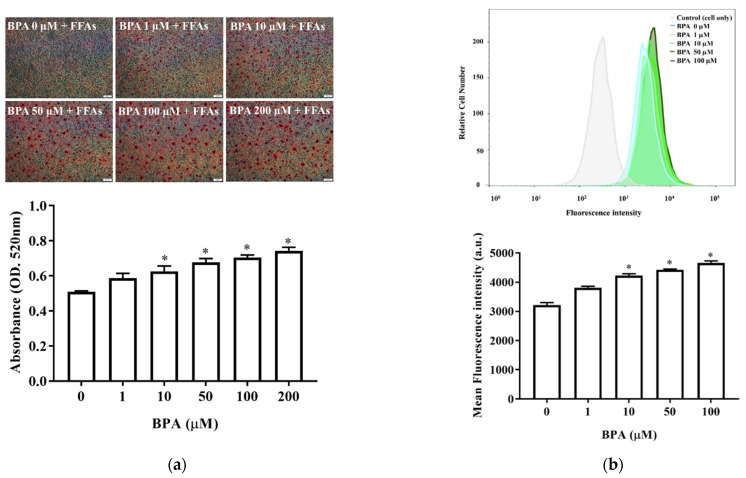
BPA increases hepatic lipid accumulation and fatty acid uptake: (**a**) Cells were treated with different doses of BPA plus FFAs (0.5 mM). Lipid droplets were stained using Oil Red O. The quantity of dye extracted from the stained cells was measured using spectrophotometry (below); (**b**) fatty acid uptake was determined using flow cytometry after exposure to BPA, in the absence or presence of LacCer, for 6 h. The histogram shows the mean of the fluorescence intensity results (below). The results are expressed as the mean ± SD of six independent experiments. * *p* < 0.05 vs. control (without BPA) group.

We next assessed the effects of BPA on hepatic lipid accumulation and fatty acid uptake. Oil Red O staining revealed that BPA plus FFA exposure increased intracellular lipid accumulation in HUH-7 cells in a BPA-dose-dependent manner (Figure 2a). The fluorescence lipid-labeling experiments showed that exposure to 10 µM BPA for 6 h increased intracellular fatty acid uptake in HUH-7 cells (Figure 2b). Therefore, BPA exposure increased fatty acid uptake and intracellular lipid accumulation.

### 3.3. N-Acetylcysteine (NAC) Suppresses BPA-Induced Fatty Acid Uptake and Lipid Accumulation

**Figure 3:**
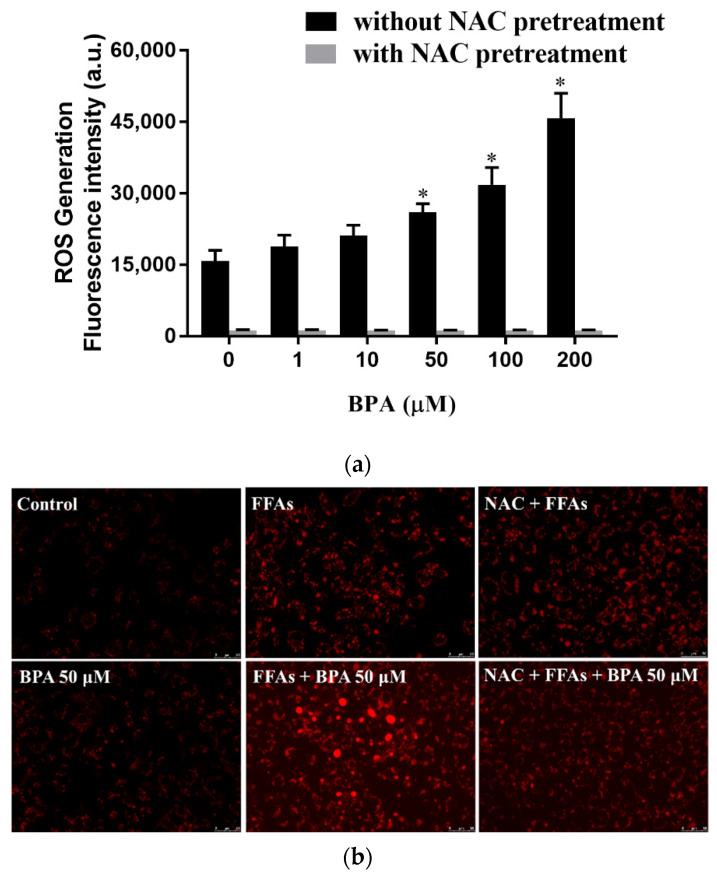

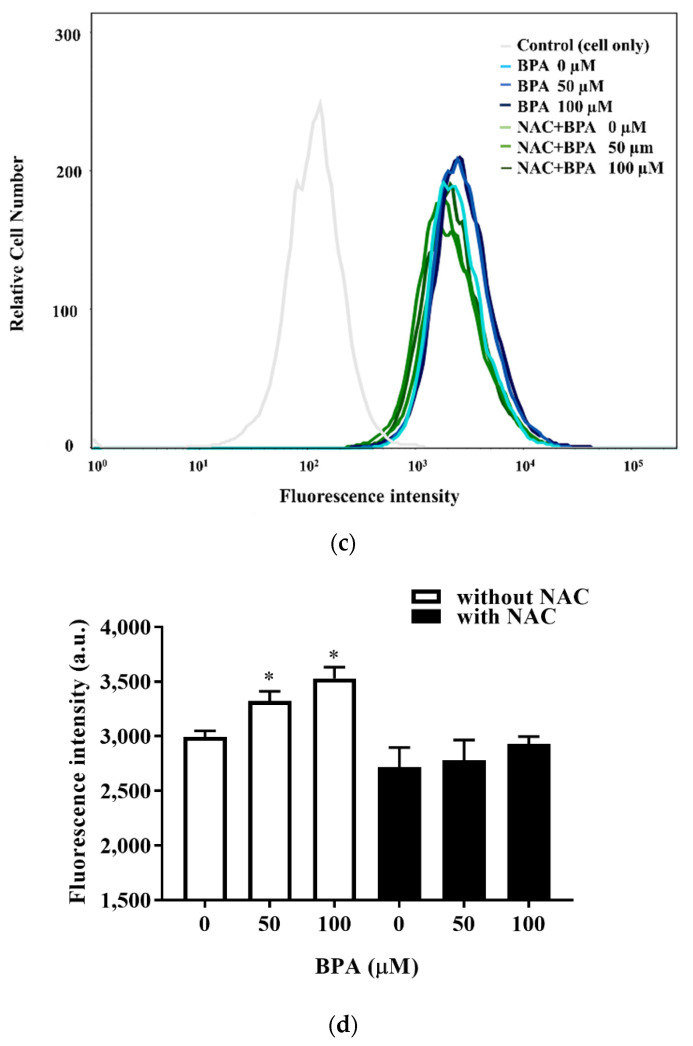
Regulation of ROS levels ameliorated BPA-induced fatty acid uptake and lipid droplet deposition in HUH-7 cells: (**a**) Cells were pretreated with 2′, 7′-dichlorofluorescin diacetate and NAC (3 mM) for 45 min, before being loaded with the indicated concentrations of BPA for 6 h. Intracellular ROS levels were determined based on fluorescence intensity; (**b**) cells were treated with BPA after pretreatment with FFAs (0.5 mM) or NAC (3 mM). The accumulation of lipid droplets was assessed using AdipoRed staining; (**c**) fatty acid uptake was determined using flow cytometry after exposure to BPA (50 μM) for 6 h, in the absence or presence of NAC pretreatment; (**d**) the mean of the fluorescence intensity results (bottom right). The results are expressed as the mean ± SD of six independent experiments. * *p* < 0.05 vs. control (without BPA and NAC) group.

We blocked intracellular free radical production with NAC to evaluate the role of ROS in the BPA-induced uptake and intracellular accumulation of lipid droplets. NAC prevented BPA-induced ROS production (Figure 3a) and, consequently, reduced BPA-induced lipid accumulation (Figure 3b). The effect of BPA exposure on intracellular fatty acid uptake was confirmed with flow cytometry using fluorescence-labeled lipids (Figure 3c,d).

### 3.4. BPA Induced Fatty Acid Uptake by Modulating CD36 Expression

**Figure 4:**
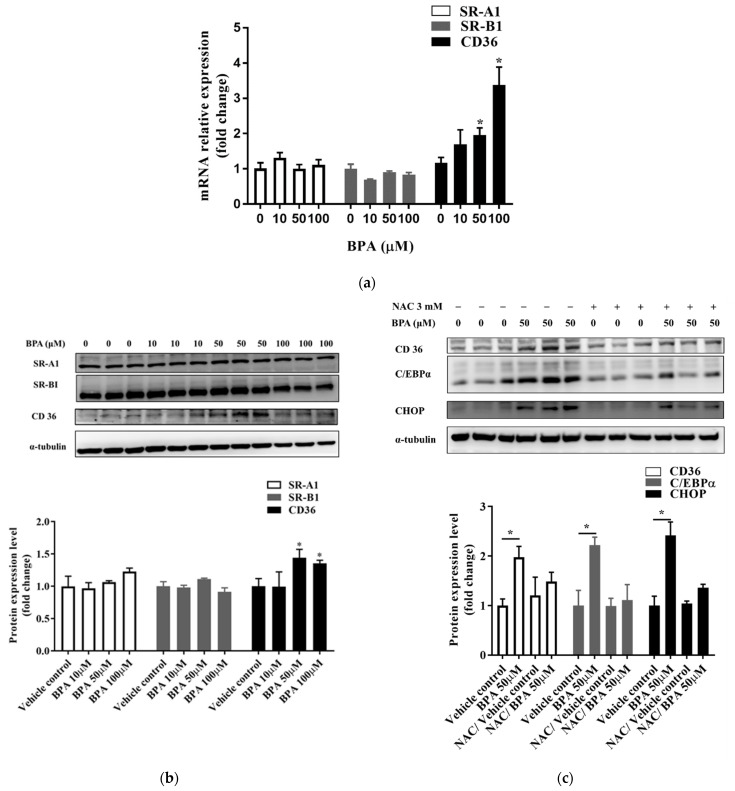
NAC inhibited the C/EBPα and CD36 lipid-related endocytosis pathway, which was upregulated by BPA exposure. Cells were treated with the indicated concentrations of BPA for 24 h. The (**a**) mRNA and (**b**) protein expression levels of CD36, SR-A1, and SR-B1 were assessed using RT-qPCR and immunoblotting, respectively. (**c**) To determine whether BPA exposure increases CD36 expression by increasing ROS production, cells were pretreated with or without NAC (3 mM) for 1 h. The protein expression levels of CD36, C/EBPα, and CHOP were determined using immunoblotting. Protein expression levels were normalized to α-tubulin. Data are shown as the mean ± SD of three independent experiments. * *p* < 0.05 vs. vehicle control group.

BPA exposure caused a significant increase in the mRNA and protein expression levels of CD36, but did not affect the expression of SR-A1 or SR-B1 (Figure 4a,b and Figure S1a,b). We assessed whether NAC could prevent BPA-induced ROS production and ER stress. Results showed that the expression of CHOP decreased in cells pretreated with NAC (Figure 4c and Figure S2a,b). In addition, NAC pretreatment attenuated the BPA-induced expression of CD36 and CCAAT-enhancer-binding protein α (C/EBPα). These results confirm the direct effect of BPA-induced ROS production on intracellular fatty acid uptake.

### 3.5. BPA Enhanced Hepatic Pathological Progression in Mice

**Figure 5:**
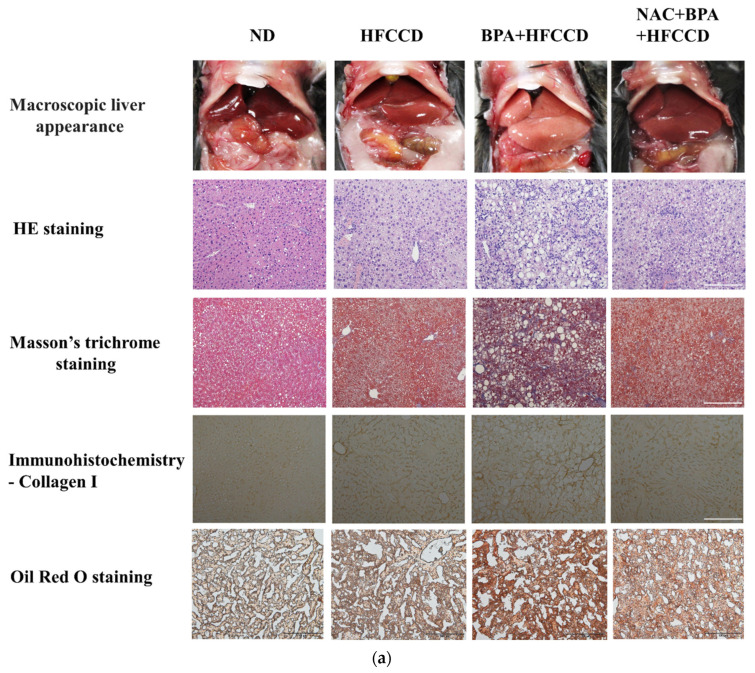

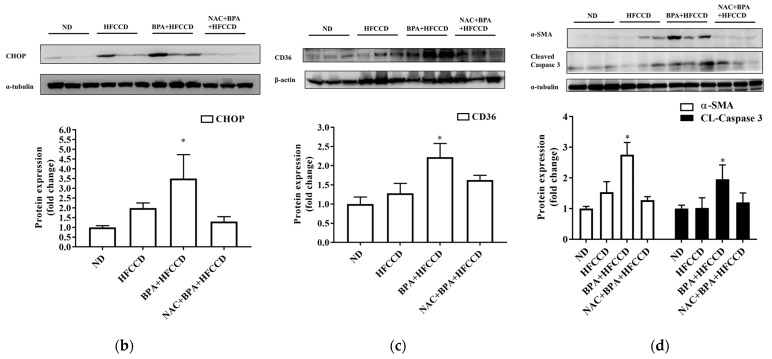
Regulation of ROS levels attenuated BPA-induced hepatic lipid accumulation and liver fibrosis. Eight-week-old C57BL/6 mice were administered an ND, HFCCD, BPA + HFCCD (50 μg/kg/day), or NAC (1 mg/mL, dissolved in drinking water) + BPA (50 μg/kg/day) + HFCCD for 8 weeks: (**a**) Images of liver tissues isolated from 8-week-old mice fed an ND, HFCCD, BPA + HFCCD, or NAC + BPA + HFCCD for 8 weeks, respectively. Sections of liver tissue were exposed to H&E staining, Masson’s trichrome staining (collagen fibers stained blue), Oil red O staining, and immunohistochemical staining (collagen 1). Scale bar: 200 μm. Protein expression of (**b**) CHOP, (**c**) CD36, and (**d**) α-SMA and cleaved caspase 3 (CL-Caspase3) in liver tissue was measured using western blot. Protein expression levels were normalized to α-tubulin. Data are shown as the mean ± SD of three independent experiments. * *p* < 0.05 vs. ND group.

We investigated the effect of BPA exposure on the progression of NAFLD in mice administered a high-fat diet. The average daily food and water intake of mice in the ND, HFCCD, BPA + HFCCD, and NAC + BPA + HFCCD groups were 2.9, 2.6, 2.4, and 2.6 g, and 4.6, 4.3, 4.2, and 4.3 mL, respectively. The liver and spleen weights, as well as their proportion of the body weight, were higher in the BPA + HFCCD group than in the NAC + BPA + HFCCD group (Table 1). The plasma levels of ALT, insulin, and total cholesterol, as well as the total cholesterol content of the liver, were significantly higher in the BPA + HFCCD group than in the NAC + BPA + HFCCD group (Table 2). According to the level of 8-OHdG (an indicator of oxidative DNA damage) in the liver tissue, NAC acted as an antioxidant and prevented liver damage caused by BPA-induced oxidative stress (Table 2). Furthermore, the livers of the BPA + HFCCD group had a paler color and showed inflammation (H&E staining), hepatosteatosis (Masson’s trichrome staining), hepatic lipid accumulation (Oil red O staining), and liver fibrosis (collagen I) (Figure 5a). These histological features were eliminated by treatment with NAC. The levels of CHOP (Figure 5b and Figure S3a) and CD36 (Figure 5c and Figure S3b) were significantly higher in liver tissue after BPA exposure and were attenuated by NAC treatment. We also determined the level of cleaved caspase 3, which would increase during the progression of NAFLD and eventually lead to liver fibrosis. Cleaved caspase 3 levels were significantly upregulated by BPA and downregulated by NAC (Figure 5d and Figure S3d). α-SMA (a fibrosis marker) levels were increased by BPA and ameliorated by NAC treatment (Figure 5d and Figure S3c). These results suggest that BPA-mediated HFCCD promotes hepatic pathological progression, resulting from ROS-induced free fatty acid uptake and lipid accumulation.

## 4. Discussion

Some recent studies have highlighted the link between NAFLD and endocrine-disruptive chemicals, such as BPA. BPA can produce hepatosteatosis in human hepatocytes by upregulating the endocannabinoid system [29]. Furthermore, BPA plays a role in the onset and progression of NAFLD through its pleiotropic action on key pathophysiological factors [30]. The mechanism of action of BPA on the induction and progression of NAFLD remains unclear. Therefore, the present study used long-chain fatty acids to investigate the effect of BPA in exacerbating NAFLD. Our in vitro experiments indicated that BPA increased fatty acid uptake and lipid accumulation by enhancing C/EBPα and CD36 protein levels as a result of increased ROS production. Moreover, the in vivo study revealed that blocking the generation of ROS attenuated the effects of BPA on NAFLD progression. These results suggest that BPA and a HFCCD can stimulate the accumulation of lipids in hepatocytes, induce ROS production, and promote the progression of NAFLD. The presence of oxidative stress has been associated with NAFLD/non-alcoholic steatohepatitis (NASH) [31,32]. BPA reportedly promotes the generation of intracellular peroxides and mitochondrial superoxide, the risk of cardiovascular diseases [33], and the progression of diabetes, obesity, and cancer [34,35,36].

Carchia et al. reported that BPA exposure caused a time-dependent decrease in mitochondrial membrane potential and increased cellular ROS levels, leading to the induction of cell apoptosis [37]. In this study, BPA exposure dramatically decreased cell viability (Figure 1a) and increased ROS generation in HUH-7 cells (Figure 1b). Free fatty acids are major mediators of hepatic lipid deposition. Free fatty acid uptake and lipid accumulation in hepatocytes were significantly increased after BPA exposure (Figure 2a,b), which suggests that BPA exposure may contribute to NAFLD progression through BPA-mediated free fatty acid uptake and lipid accumulation.

NAFLD is a complex disease caused by both genetic and environmental factors, e.g., endocrine-disrupting chemicals (EDCs); however, a comprehensive understanding of the independent mechanisms of NAFLD pathogenesis remains lacking. Exposure to various EDCs, such as BPA, has been associated with hepatic lipid accumulation and metabolic disorders [38]. Some studies have linked BPA-induced oxidative stress to the promotion of NASH and hepatocellular carcinoma (HCC) [10,39,40]. BPA exposure increases the binding of Nrf2 to a putative antioxidant-response-element consensus sequence in the Srebp-1c promoter and increases fatty acid and lipid production [14]. In the present study, we observed significant cellular damage after BPA exposure in vitro (Figure 1a) and in vivo (Figure 5d). NAC pretreatment attenuated the adverse effects induced by BPA (e.g., cytotoxicity and ROS production) both in vitro and in vivo (Figure 3 and Figure 5). 8-OHdG is a useful biomarker for oxidative DNA damage and has been reported as a feature of carcinogenesis in several studies [41,42]. The concentration of oxidative stress index 8-OHdG in the liver tissue of the BPA + HFCCD group was higher than that of the NAC + BPA + HFCCD group. The inhibitory action of NAC attenuated the tissue damage resulting from BPA-induced ROS production. These results suggest that BPA mediates free-fatty-acid-induced hepatosteatosis through the production of ROS.

Seo et al. [26] demonstrated that ROS production could increase the intracellular uptake of glucose and cholesterol. SRs, such as CD36, SR-A1, and SR-B1, are known to participate in the uptake and efflux of cholesterol, and contribute to lipid accumulation and metabolic dysfunction in conditions of excessive fat supply [43]. ER stress responses play a critical role in the CD36-mediated uptake of oxidized LDLs in macrophages [44]. We found, in the in vitro experiment, that BPA induced ROS production which, in turn, enhanced CD36 expression and fatty acid uptake. NAC is a commonly used antioxidant that can effectively inhibit ROS reactivity. In preclinical models of NAFLD, NAC blocked hepatic lipid accumulation, which indicates that NAC can regulate fatty acid scavenger molecule CD36 and transcriptional factors, such as Srebp-1c [45]. In this study, NAC pretreatment attenuated the effects of BPA-induced ER stress as well as the upregulation of CD36, which is responsible for the increase in fatty acid uptake (Figure 4c and Figure 5c). Collectively, these findings demonstrate that BPA-induced ROS production and the scavenging mechanisms of CD36 overexpression could lead to hepatic lipid accumulation.

Most food containers (e.g., canned food and plastic or paper packaging) contain BPA, and its transfer to food from these containers has been reported, which stokes legitimate health concerns [46,47,48]. Based on the highest observed degree of BPA migration from acidic- and fatty-food containers, daily BPA exposure in humans is predicted to be <1 μg/kg body weight/day [47,48]. The US Environmental Protection Agency reported that the lowest observed adverse effect level (LOAEL) for oral exposure to BPA in rodents is 50 mg/kg/day [49]. Although daily exposure in humans is lower than the LOAEL reported for rodents, we selected 50 μg/kg/day BPA in the present study based on the concentration ranges reported in other animal experiments [50,51].

We combined a HFCCD with BPA in the present study to simulate a high-fat human diet coupled with environmental toxicant exposure. A more serious stage than NAFLD called nonalcoholic steatohepatitis (NASH) can cause severe liver damage and liver failure, leading to rapid body-weight loss [52]. In this study, we observed a relative increase in liver and spleen weight, but a significant decrease in the body weight of the mice fed BPA+HFCCD for 8 weeks. A high-fat/high-cholesterol diet has been reported to increase liver weight, as well as fat infiltration, triglycerides, and total cholesterol in the liver, compared with an ND and HFD diet [53]. Furthermore, it also has been indicated that administration of a methionine-choline-deficient (MCD) diet to mice induces a rapid and severe steatohepatitis state. Compared to the mice fed the control diet for the same duration, mice lost body weight after receiving the MCD diet [54]. We found that the markers of hepatosteatosis (Masson’s trichrome staining), as well as collagen I expression (IHC staining), were higher in the BPA + HFCCD group compared with the other groups (Figure 5a). The protein expression of cleaved caspase 3 was also higher in the BPA + HFCCD group, which implies that exposure to BPA and a high-fat diet may increase the progression of NAFLD and, eventually, lead to liver fibrosis. NAC downregulated the protein expression of CHOP, CD36 (Figure 3b,c), and cleaved caspase 3 (Figure 5d). These results suggest that BPA exposure coupled with a HFCCD induces hepatosteatosis through ROS-induced CD36 overexpression, as well as hepatic lipid accumulation.

**Figure 6:**
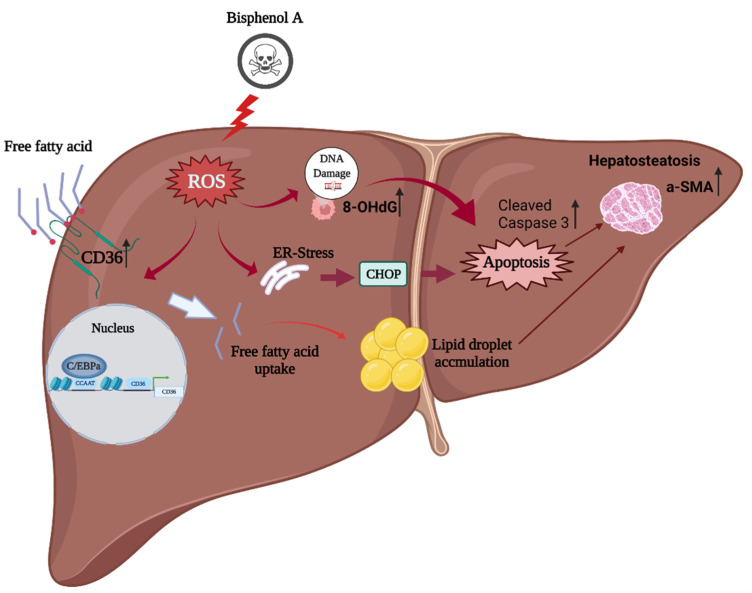
The proposed mechanism of BPA coupled with a high-fat diet in enhancing ROS-induced hepatic lipid accumulation and liver fibrosis: BPA + HFCCD increases intracellular ROS production, which induces the expression of CCAAT-enhancer-binding protein α (C/EBPα) and CD36, and promotes free fatty acid uptake. BPA exposure aggravates HFCCD-induced liver damage, leading to cleaved caspase-3 activation for apoptosis, steatohepatitis, and acceleration of the fibrotic process (due to α-SMA overexpression). The figure was created using Biorender.

To summarize: BPA exposure in a high-fat-diet model increased intracellular ROS production which, in turn, induced C/EBPα and CD36 overexpression to promote intracellular free fatty acid uptake, liver damage, and caspase-3 activation for apoptosis; increased α-SMA expression for steatohepatitis; and accelerated the fibrotic process (Figure 6). However, the detailed mechanism by which BPA induces ROS production to increase CD36 expression and free fatty acid accumulation requires further research.

## 5. Conclusions

This study demonstrates that BPA exposure may increase ROS production and CD36 expression, which promotes intracellular free fatty acid uptake and leads to liver damage and caspase-3 activation. The increased levels of α-SMA expression with BPA exposure can potentially accelerate steatohepatitis and the fibrotic process. Therefore, it is necessary for consumers to pay attention to the containers used in the storage of high-fat foods to minimize the adverse health effects of BPA.

## Figures and Tables

**Table 1:** Effects of bisphenol A (BPA) on the growth characteristics of mice administered a high-fat/high-cholesterol/high-cholic-acid diet (HFCCD).

	ND	HFCCD	BPA + HFCCD	NAC + BPA + HFCCD
Initial body wt (g)	24.0 ± 1.3	23.6 ± 0.6	24.0 ± 1.7	24.0 ± 0.5
Body wt after 4 weeks of the HFCCD diet (g)	27.2 ± 1.2 ^b^	27.2 ± 0.9 ^b^	29.5 ± 1.8 ^a^	26.2 ± 0.5 ^b^
Final body wt (g)	28.7 ± 2 ^b^	28.8 ± 1.3 ^b^	25.9 ± 0.4 ^a^	28.1 ± 1.6 ^b^
Liver wt (g)	1.046 ± 0.027 ^c^	1.301 ± 0.073 ^b^	1.549 ± 0.135 ^a^	1.403 ± 0.091 ^b^
Liver wt/ body wt (%)	3.665 ± 0.239 ^c^	4.539 ± 0.416 ^b^	6.004 ± 0.434 ^a^	4.997 ± 0.224 ^b^
Spleen wt (g)	0.077 ± 0.006 ^c^	0.099 ± 0.005 ^b^	0.117 ± 0.002 ^a^	0.098 ± 0.010 ^b^
Spleen wt/ body wt (%)	0.268 ± 0.023 ^c^	0.346 ± 0.028 ^b^	0.452 ± 0.016 ^a^	0.350 ± 0.052 ^b^
Epididymal fat wt (g)	0.751 ± 0.089	0.795 ± 0.075	0.864 ± 0.271	0.662 ± 0.091
Epididymal fat wt/ body wt (%)	2.646 ± 0.464	2.761 ± 0.178	3.353 ± 1.062	2.351 ± 0.239
Brown fat wt (g)	0.119 ± 0.019 ^a^	0.112 ± 0.009 ^a^	0.078 ± 0.016 ^b^	0.108 ± 0.009 ^a^
Brown fat wt/ body wt (%)	0.415 ± 0.065 ^a^	0.390 ± 0.028 ^a^	0.302 ± 0.028 ^b^	0.381 ± 0.072 ^a^

Data are presented as the mean ± SD; ^a,b,c^ *p* < 0.05.

**Table 2:** Effects of BPA on blood and hepatic biochemical parameters in mice administered an HFCCD.

	ND	HFCCD	BPA + HFCCD	NAC + BPA + HFCCD
***Serum***				
ALT (U/L)	26.20 ± 4.21 ^b^	63.40 ± 15.82 ^a^	93.80 ± 34.58 ^a^	52.60 ± 11.90 ^a^
Triglycerides (mg/dL)	69.4 ± 7.83 ^a^	19.20 ± 4.32 ^b^	42.00 ± 12.14 ^a^	73.40 ± 32.21 ^a^
Total cholesterol (mg/dL)	134.40 ± 13.83 ^b^	164.40 ± 27.93 ^b^	280.20 ± 35.72 ^a^	131.20 ± 16.96 ^b^
Blood glucose (mg/dL)	152.67 ± 33.63	148.00 ± 5.93	155.00 ± 18.81	143.17 ± 7.60
Insulin (ng/mL)	0.177 ± 0.081 ^c^	0.418 ± 0.13 ^b^	0.651 ± 0.205 ^a^	0.374 ± 0.091 ^b^
HOMA	1.421 ± 0.453 ^c^	3.466 ± 1.023 ^b^	5.717 ± 2.169 ^a^	3.027 ± 0.774 ^b^
***Liver***				
Triglycerides (mg/g of tissue)	43.17 ± 9.66	57.36 ± 12.52	55.44 ± 10.40	47.01 ± 8.06
Total cholesterol (mg/g of tissue)	6.12 ± 0.74 ^c^	51.74 ± 10.18 ^b^	70.50 ± 5.44 ^a^	50.8 ± 10.81 ^b^
8-OHdG (ng/g of tissue)	0.577 ± 0.135 ^c^	1.158 ± 0.148 ^b^	2.207±0.219 ^a^	1.224 ± 0.281 ^b^

Data are presented as the mean ± SD; ^a,b,c^ *p* < 0.05.

## Supplementary Materials

The following supporting information can be downloaded at: https://www.mdpi.com/article/10.3390/toxics10050208/s1, Figure S1: (a) Original unedited blot, ECL images indicating SR-A1, SR-B1 and α-tubulin for representative Western blots used in Figure 4b of the manuscript. (b) Original unedited blot, ECL images indicating CD36 and α-tubulin for representative Western blots used in Figure 4b of the manuscript; Figure S2: Original unedited blot, ECL images indicating CD36, C/EBPα, CHOP and α-tubulin for representative Western blots used in Figure 4c of the manuscript; Figure S3: (a) Original unedited blot, ECL images indicating CHOP and α-tubulin for representative Western blots used in Figure 5b of the manuscript. (b) Original unedited blot, ECL images indicating CD36 and β-actin for representative Western blots used in Figure 5c of the manuscript. (c) Original unedited blot, ECL images indicatingα-SMA and α-tubulin for representative Western blots used in Figure 5d of the manuscript. (d) Original unedited blot, ECL images indicating CL-caspase 3 and α-tubulin for representative Western blots used in Figure 5d of the manuscript.
