# Supplementary material for: Bisphenol A Coupled with a High-Fat Diet Promotes Hepatosteatosis through Reactive-Oxygen-Species-Induced CD36 Overexpression
Source: Toxics. 2022 Apr 22;10(5):208. doi: 10.3390/toxics10050208 (PMC9145332; doi:10.3390/toxics10050208)
Supplement: Supplementary file 1 [file toxics-10-00208-s001.zip › toxics-1650813-supplementary.pdf]

toxics

Supplementary Materials: Bisphenol A Coupled with a High-Fat Diet Promotes Hepatosteatosis through Reactive-Oxygen-Species-Induced CD36 Overexpression

Jyun-Lin Lee, Yao-Chien Wang, Yu-An Hsu, Chih-Sheng Chen, Rui-Cian Weng, Yen-Pei Lu, Chun-Yu Chuang and Lei Wan

Original Images for Blots

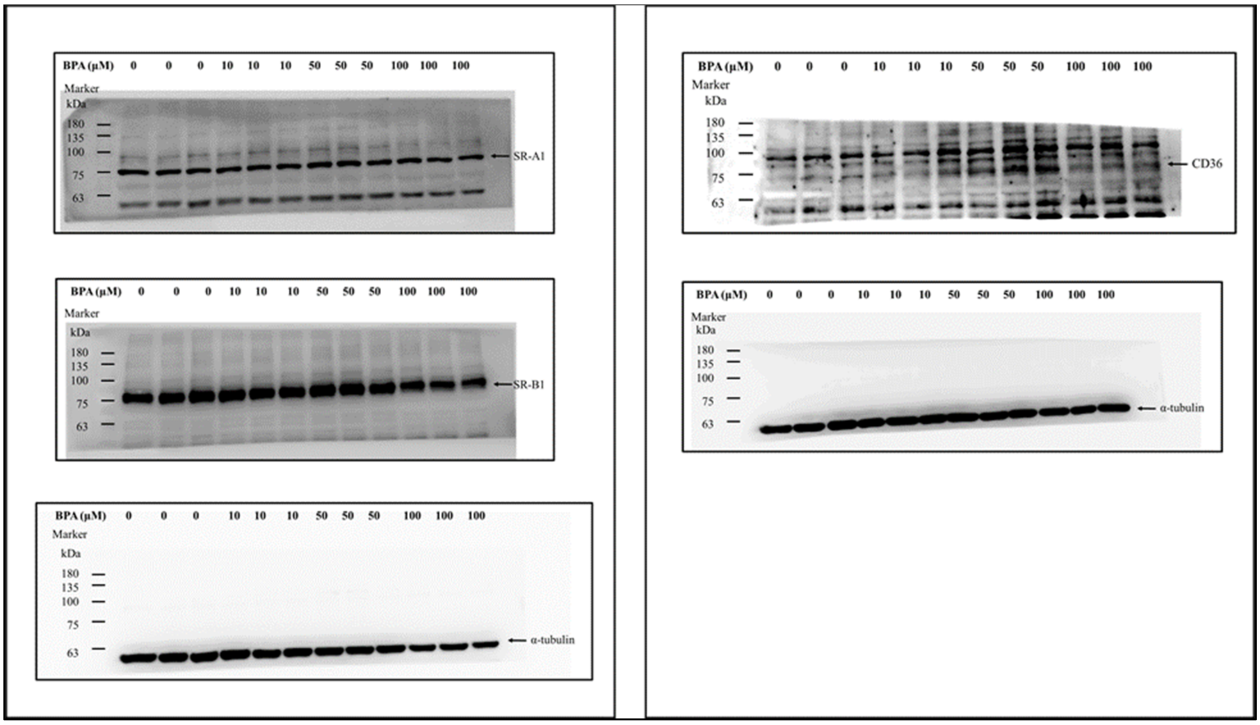

(a) (b)

Figure S1. (**a**) Original unedited blot, ECL images indicating SR-A1, SR-B1 and α-tubulin for representative Western blots used in Fig. 4b of the manuscript. (**b**) Original unedited blot, ECL images indicating CD36 and α-tubulin for representative Western blots used in Fig. 4b of the manuscript.

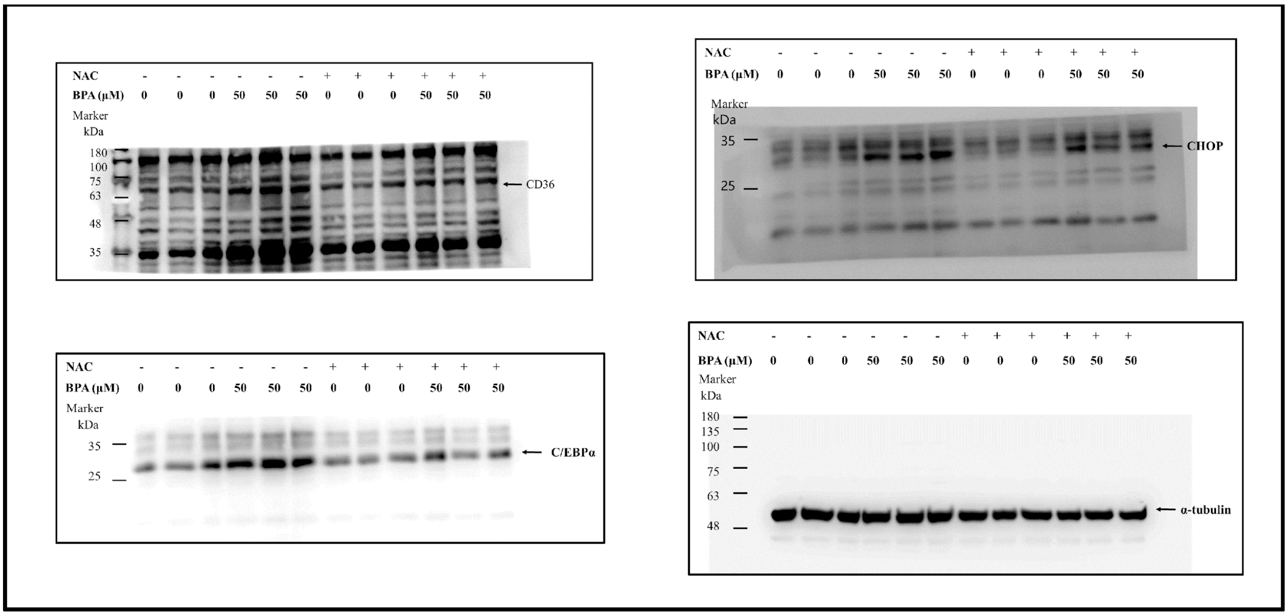

Figure S2. Original unedited blot, ECL images indicating CD36, C/EBPα, CHOP and α-tubulin for representative Western blots used in Fig. 4c of the manuscript.

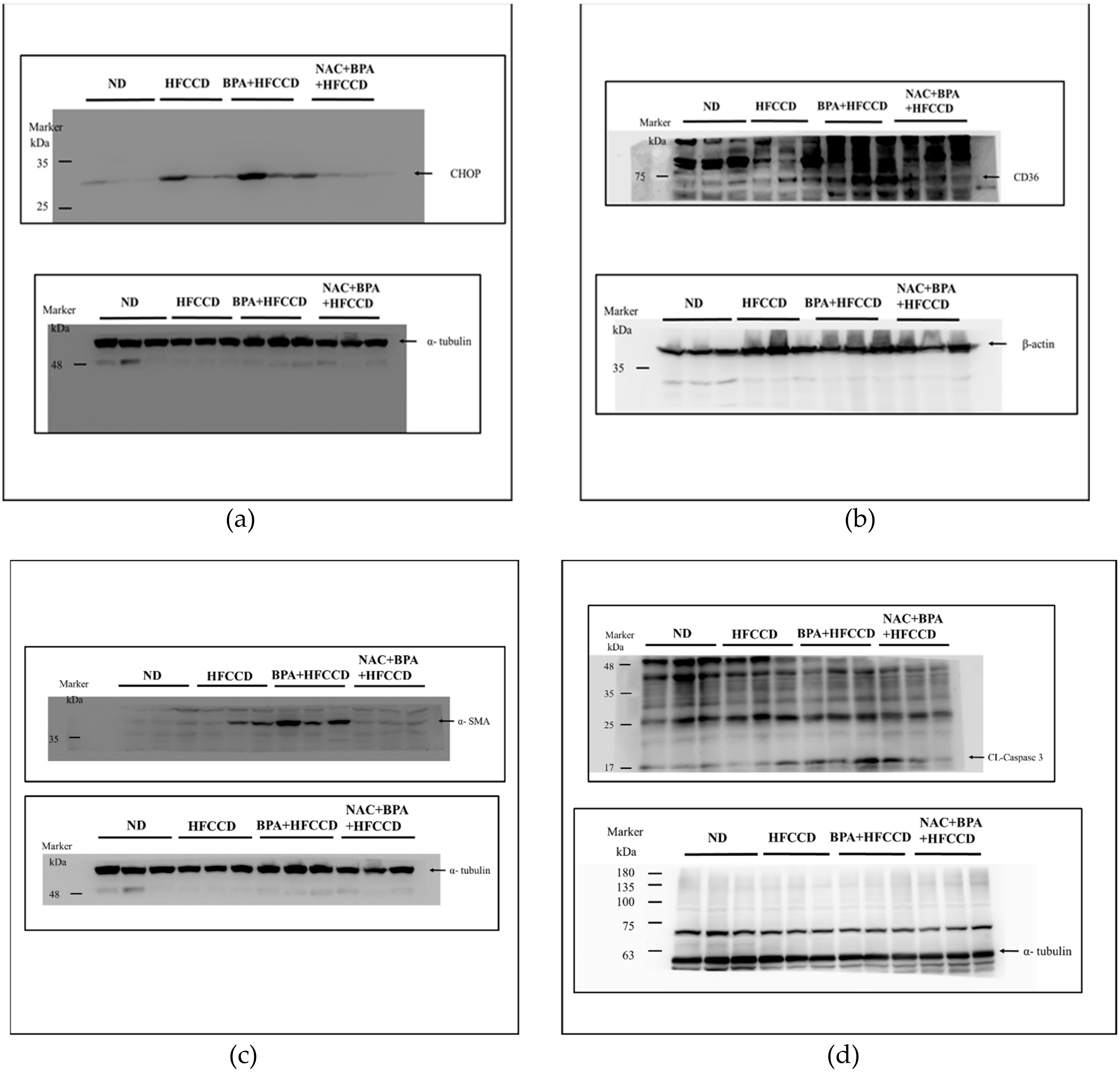

Figure S3. (**a**) Original unedited blot, ECL images indicating CHOP and α-tubulin for representative Western blots used in Fig. 5b of the manuscript. (**b**) Original unedited blot, ECL images indicating CD36 and β-actin for representative Western blots used in Fig. 5c of the manuscript. (**c**) Original unedited blot, ECL images indicatingα-SMA and α-tubulin for representative Western blots used in Fig. 5d of the manuscript. (**d**) Original unedited blot, ECL images indicating CL-caspase 3 and α-tubulin for representative Western blots used in Fig. 5d of the manuscript.
